# Identification of the source of elevated hepatocyte growth factor levels in multiple myeloma patients

**DOI:** 10.1186/2050-7771-2-8

**Published:** 2014-04-09

**Authors:** Christoph Rampa, Erming Tian, Thea Kristin Våtsveen, Glenn Buene, Tobias Schmidt Slørdahl, Magne Børset, Anders Waage, Anders Sundan

**Affiliations:** 1 The K. G. Jebsen Center for Myeloma Research and Department of Cancer Research and Molecular Medicine, Norwegian University of Science and Technology, Trondheim, Norway; 2 Section of Hematology, St. Olavs University Hospital, Trondheim, Norway; 3 The Donna D. and Donald M. Lambert Laboratory of Myeloma Genetics, Myeloma Institute for Research and Therapy, University of Arkansas for Medical Sciences, Little Rock, Arkansas, USA

**Keywords:** Multiple myeloma, Hepatocyte growth factor, Scatter factor, Bone marrow core biopsies, Microarray, Fluorescence *in situ* hybridization, DNA sequencing, Co-cultivation

## Abstract

**Background:**

Hepatocyte growth factor (HGF) is a pleiotropic cytokine which can lead to cancer cell proliferation, migration and metastasis. In multiple myeloma (MM) patients it is an abundant component of the bone marrow. HGF levels are elevated in 50% of patients and associated with poor prognosis. Here we aim to investigate its source in myeloma.

**Methods:**

HGF mRNA levels in bone marrow core biopsies from healthy individuals and myeloma patients were quantified by real-time PCR. *HGF* gene expression profiling in CD138^+^ cells isolated from bone marrow aspirates of healthy individuals and MM patients was performed by microarray analysis. HGF protein concentrations present in peripheral blood of MM patients were measured by enzyme-linked immunosorbent assay (ELISA). Cytogenetic status of CD138^+^ cells was determined by fluorescence *in situ* hybridization (FISH) and DNA sequencing of the *HGF* gene promoter. HGF secretion in co-cultures of human myeloma cell lines and bone marrow stromal cells was measured by ELISA.

**Results:**

HGF gene expression profiling in both bone marrow core biopsies and CD138^+^ cells showed elevated HGF mRNA levels in myeloma patients. HGF mRNA levels in biopsies and in myeloma cells correlated. Quantification of HGF protein levels in serum also correlated with HGF mRNA levels in CD138^+^ cells from corresponding patients. Cytogenetic analysis showed myeloma cell clones with *HGF* copy numbers between 1 and 3 copies. There was no correlation between *HGF* copy number and HGF mRNA levels. Co-cultivation of the human myeloma cell lines ANBL-6 and JJN3 with bone marrow stromal cells or the HS-5 cell line resulted in a significant increase in secreted HGF.

**Conclusions:**

We here show that in myeloma patients HGF is primarily produced by malignant plasma cells, and that HGF production by these cells might be supported by the bone marrow microenvironment. Considering the fact that elevated HGF serum and plasma levels predict poor prognosis, these findings are of particular importance for patients harbouring a myeloma clone which produces large amounts of HGF.

## Introduction

Multiple Myeloma (MM) is a neoplasm of terminally differentiated antibody-producing B-cells [1]. Malignant plasma cells (PC) are, except for in very late stages of disease, predominantly found within the bone marrow, and the cells are believed to depend on the bone marrow microenvironment for survival. Malignant PCs interact with and may modify their microenvironment leading to altered cytokine secretion, cell homing, cell maturation and differentiation [2,3].

Hepatocyte growth factor (HGF) is a pleiotropic cytokine capable of inducing mitogenesis and morphogenesis in target cells by activation of its transmembrane receptor tyrosine kinase c-MET. In myeloma, HGF-c-MET signaling was reported to induce myeloma cell proliferation and survival [4,5]. We and others have earlier reported that about 50% of myeloma patients have elevated serum levels of HGF [6,7]. Furthermore, levels of HGF are higher in the bone marrow than in peripheral blood [6,8,9]. Importantly, elevated HGF levels predict a poor prognosis, short-term responses to therapies and early relapses [6,9,10].

Under normal conditions, HGF and c-MET are primarily expressed by mesenchymal and epithelial cells, respectively, representing an important signaling pathway for mesenchymal-epithelial interaction. However, hematopoietic cells such as B-cells are also capable of expressing both HGF and c-MET, but the expression is depending on stage of cell maturation, and results in either c-MET or HGF expression [11,12]. We have earlier shown that myeloma cell lines as well as primary myeloma cells often significantly overexpress HGF [13,14]. This, together with the fact that myeloma cells frequently co-express c-MET, suggests the presence of an autocrine signaling loop, which could promote the survival and proliferation of myeloma cells [13,15,16].

High HGF levels found in the blood and bone marrow of myeloma patients could either be the result of *HGF* overexpression in malignant PCs or due to a reactive process within the bone marrow which is a result of the presence of malignant PCs. Since the origin of excess HGF in myeloma patients is still unknown, we hypothesized that the bulk of HGF found in myeloma patients is produced by malignant PCs, and not by the bone marrow microenvironment. We therefore performed experiments which were aimed at identifying the source of excess HGF. In summary, we show by microarray, real-time PCR, fluorescence *in situ* hybridization, Sanger DNA sequencing and co-cultivation experiments that in patients with very high serum levels of HGF protein, malignant PCs and not the bone marrow microenvironment are responsible for excess HGF production. Furthermore, serum HGF reflects overexpression of HGF in the malignant PCs.

## Methods

### Patient samples

Samples used in this study comprised blood sera from multiple myeloma patients, bone marrow aspirates taken from healthy individuals and from patients suffering from different stages of disease as defined based on the International Myeloma Working Group consensus guidelines and bone marrow core biopsies isolated from healthy individuals and MM patients [17]. Human myeloma cell lines (HMCL) were also included in this study.

Serum samples were taken at diagnosis and before the initiation of treatment. Bone marrow aspirates and bone marrow core biopsies were taken from the left or right posterior superior iliac crest at diagnosis before treatment was initiated using established surgical procedures at the University of Arkansas Medical Sciences, Little Rock, Arkansas, USA or at the Department of Hematology/Regional Research Biobank of Central Norway, St. Olavs University Hospital, Trondheim, Norway. Plasma cells were purified from bone marrow aspirates by CD138^+^ magnetic-activated cell sorting (MACS) Microbeads (Miltenyi, Auburn, CA, USA) essentially as described elsewhere [18]. The bone marrow core biopsies of the patients with MM were divided into two portions, with one portion instantaneously submerged in liquid nitrogen for total RNA extraction and the other preserved in a fixative, and then embedded in paraffin for histological examination (n = 46). The paraffin-biopsy materials were sectioned and stained with hematoxylin-eosin, Giemsa, and Prussian blue. Trained pathologists estimated the fraction of PCs in the bone marrow biopsies.

Samples were collected after informed consent was given by the patients. An institutional review board-approved consent form, which was in accordance with the Declaration of Helsinki, was used to receive patient consent. The study was approved by the Norwegian Regional Ethics Committee (REK 2011–2029), and by the Institutional Review Board of the University of Arkansas for Medical Sciences.

### Nucleic acid preparations

Genomic DNA and/or total RNA was isolated from normal PCs, primary myeloma PCs and myeloma cell lines (0.5 to 5.0 × 10^6^ cells) using the AllPrep DNA/RNA Mini Kit (Qiagen, Valencia, CA, USA). The RNeasy Fibrous Tissue Kit (Qiagen) was used to extract total RNA from ultra-low temperature (liquid nitrogen) preserved bone marrow core biopsies.

### Gene expression profiling of primary myeloma cells

Gene expression profiling was performed as previously described using the Affymetrix U133Plus2.0 microarray (Affymetrix, Santa Clara, CA, USA) [19-22]. Microarray data of the *HGF* gene expression profile in PCs isolated from 22 healthy donors (NPC), 14 patients diagnosed with monoclonal gammopathy of undetermined significance (MGUS), 34 patients with smouldering MM (SMM), 344 MM patients and 45 HMCLs were retrieved from the NIH Gene Expression Omnibus17, which can be found under accession number GSE2658. The Mann–Whitney test (two-tailed) was performed for analysis of statistical significance.

### Quantification of HGF mRNA levels in patient samples by real-time PCR

HGF mRNA levels in bone marrow core biopsies taken from 19 healthy individuals and 46 MM patients and in CD138^+^ cells purified from bone marrow aspirates of 24 MM patients were quantified by TaqMan® real-time PCR. Total RNA (1.0 μg) was reverse-transcribed using the High Capacity RNA-to-CDNA Kit (Life Technologies, Carlsbad, CA, USA), applying oligo(dT) primers. The HGF (Hs00379140_m1) TaqMan® probe was used to detect gene expression and GAPDH (Hs99999905_m1) was used as endogenous reference (Life Technologies, Carlsbad, CA, USA).

### PCR amplification and sequencing

*HGF* promoter fragments present in CD138^+^ cells purified from bone marrow aspirates from 12 MM patients were amplified from genomic DNA templates using the PfuUltra II Fusion HS DNA Polymerase (Stratagene, Santa Clara, CA, USA). To facilitate amplification, the *HGF* promoter was divided in four overlapping segments. For primers see Additional file 1: Table S1. PCR products were treated with an exonuclease I and shrimp alkaline phosphatase blend (ExoSAP-IT PCR Clean-up Kit, GE Healthcare, Waukesha, WI, USA), and directly used for sequencing reactions. Both DNA strands were sequenced using the BigDye Terminator v1.1 Cycle Sequencing Kit (Applied Biosystems, Carlsbad, CA, USA). Sequencing reactions were analyzed in a 3130x/Genetic Analyzer (Applied Biosystems).

The deoxyadenosine tract elements (DATE) present in the *HGF* promoter of CD138^+^ cells purified from bone marrow aspirates of 24 MM patients were amplified as described elsewhere [23] and sub-cloned into the pCR2.1 vector (Invitrogen, UK). Sequencing was performed on 2–3 clones from each patient using M13 standard primers.

### Fluorescence *in situ* hybridization (FISH)

FISH was performed on CD138^+^ cells purified from bone marrow aspirates of 24 MM patients. Probes for FISH were made from Bacterial Artificial Chromosome (BAC) clones (BACPAC resources, Children’s Hospital Oakland, CA, USA). BAC clones RP11-117 L18 and RP11-433O12 which are centromeric to *HGF* were labeled in SpectrumOrange and BAC clones RP11-657 J19 and RP11-451D20 which are telomeric to *HGF* were labeled in SpectrumAqua. Centromeric enumeration probe 7 in green (Vysis, Abott laboratories, Des Plaines, IL, USA) was used to assess the chromosome copy number. Sample preparation and microscopy was performed as earlier described [24,25].

### Co-cultivation of bone marrow stromal cells (BMSC) and human myeloma cell lines

Preparation of BMSC was performed as described in detail by Misund *et al.*[26]. In short, CD138^−^ bone marrow mononuclear cells were seeded in cell culture flasks, and after 3 days non-adherent cells were removed. The remaining cells were expanded for three to four weeks. Stromal cells from ten different patients were mixed to obtain a batch of standardized BMSC. Each batch of BMSC was characterized by immunophenotyping, using an LSRII flow cytometer (BD Biosciences, San Jose, CA, USA). The bone marrow stromal cells consisted essentially of fibroblast-like cells [26].

For co-cultivation experiments, BMSC or HS-5 cells [27], were seeded at a concentration of 3 × 10^4^ cells per well (0.5 mL) into 24 well plastic plates and allowed to adhere for 24 h at 37°C in a humidified atmosphere containing 5% CO_2_. Then, 2 to 6 × 10^4^ myeloma cells (0.1 mL; cell number depended on cell line used) were added and cultivation continued until supernatants were harvested after 48 h. Later, the levels of HGF in the supernatants were measured by ELISA. The cell lines HS-5 [27], U266 [28], and the human T-cell leukemia Jurkat [29] were purchased from American Type Culture Collection (ATCC, Rockville, MD, USA). ANBL-6 cells [30] and INA-6 cells [31] were kind gifts from Dr. Jelinek (Mayo Clinic, Rochester, MN, US) and Dr. Gramazki (University of Erlangen-Nuremberg, Erlangen, Germany), respectively. The cell line JJN3 [32] was a kind gift from Dr. Ball (University of Birmingham, UK). The IH-1 [33] and OH-2 [34] cell lines were established in our laboratory from pleural effusions of two myeloma patients.

### Transwell cultivation of bone marrow stromal cells (BMSC) and human myeloma cell lines

For the cultivation of BMSC with myeloma cells in transwells, 3 × 10^4^ BMSC per well (0.5 mL) were seeded into inserts of 24 transwell plastic plates (pore size of 0.4 μm) and allowed to adhere for 24 h at 37°C in a humidified atmosphere containing 5% CO_2_. Then, 6 × 10^4^ ANBL-6 cells or 2 × 10^4^ JJN3 cells per well (0.1 mL) were added to the lower chambers. Supernatants were harvested after 48 h, and the HGF levels were measured by ELISA.

### Quantification of HGF by enzyme-linked immunosorbent assay (ELISA)

HGF protein concentrations were quantified in a total of 53 blood sera taken from MM patients or cell supernatants using the DuoSet ELISA Development kit (R&D Systems, Minneapolis, MN, USA). Assay was performed according to manufacturer’s instructions.

### Statistical analyses

Results were considered statistically significant when p values were less than 0.05. Skewed variables were logarithmically transformed before entering a parametric analysis. Comparisons between groups were performed by the Mann–Whitney U test. To investigate linear correlations linear regression analysis was used.

## Results

### HGF mRNA levels in the bone marrow of healthy individuals and MM patients

**Figure 1:**
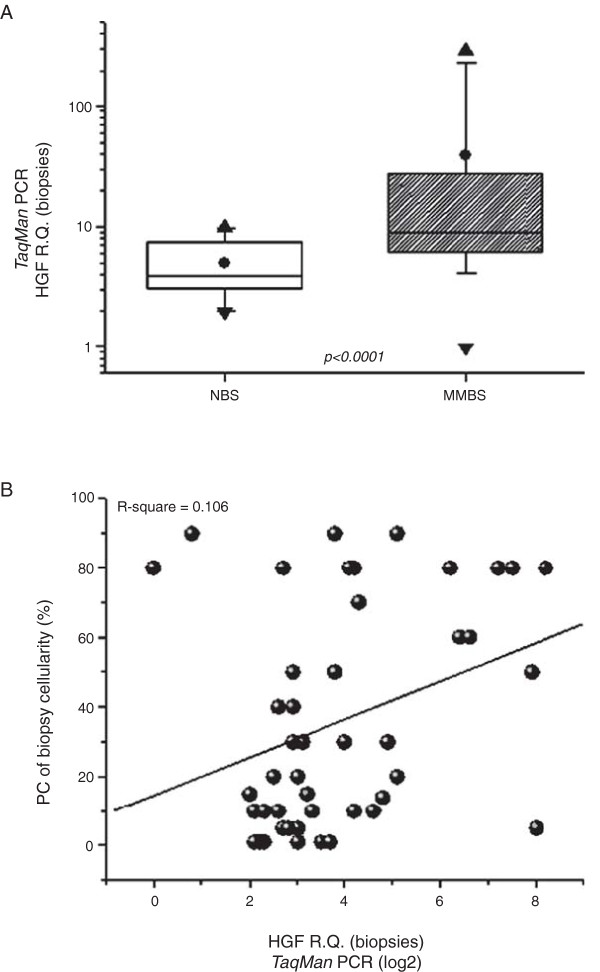
**Analysis of *HGF *gene expression in bone marrow biopsies of healthy individuals and MM patients. (A)** Quantification and statistical analysis of HGF mRNA levels in bone marrow core biopsies. HGF mRNA levels in biopsies of healthy individuals (n = 19) and myeloma patients (n = 46) were quantified by real-time PCR. The result of a two-tailed Mann–Whitney test is shown (p < 0.0001). (●) = mean, (▲) = maximum value, (▼) = minimum value, box = the distribution of the data set, and bars = standard deviation. NBS – Non-myeloma bone marrow biopsies; MMBS – Multiple myeloma bone marrow biopsies. **(B)** Linear regression analysis of HGF mRNA levels in bone marrow core biopsies aligned to the myeloma cell fraction present in the biopsies. HGF mRNA levels were quantified by real-time PCR and depicted as relative quantities (R.Q.). The proportion of malignant PCs per total cellularity of core biopsy is represented (%).

We have earlier shown that about 50% of myeloma patients have elevated HGF protein levels in the blood serum and in the bone marrow as compared to healthy individuals. However, the measured values showed considerable variation within each group [6,9,10]. Elevated HGF levels were also found in the present study for HGF mRNA in bone marrow core biopsies as shown in Figure 1A. We quantified HGF mRNA levels in biopsies of healthy individuals (NBS; n = 19) and MM patients (MMBS; n = 46) by real-time PCR. Statistical analysis (Mann–Whitney two-tailed test) indicated that the relative quantity (R.Q.) of HGF mRNA in MM biopsies (mean ± SD = 39.1 ± 69.1; range = 1.0 – 288.7) was significantly higher than that measured in healthy individuals (mean = 5.0 ± 2.4; range = 2.0 – 9.8) (p < 0.0001).

Next, the possibility that elevated HGF mRNA levels could be related to the percentage of malignant PCs present in the bone marrow was examined (Figure 1B). Linear regression analysis of the HGF mRNA levels in bone marrow core biopsies (n = 46) versus the percentage of PCs present in corresponding biopsies (n = 46) showed no significant correlation (R^2^ = 0.106). This suggests that the HGF mRNA content in bone marrow core biopsies from a group of MM patients is not associated with the proportion of myeloma cells in the bone marrow of the same patients.

### HGF expression in CD138^+^ cells isolated from bone marrow aspirates of healthy individuals and patients suffering from different stages of myeloma

**Figure 2:**
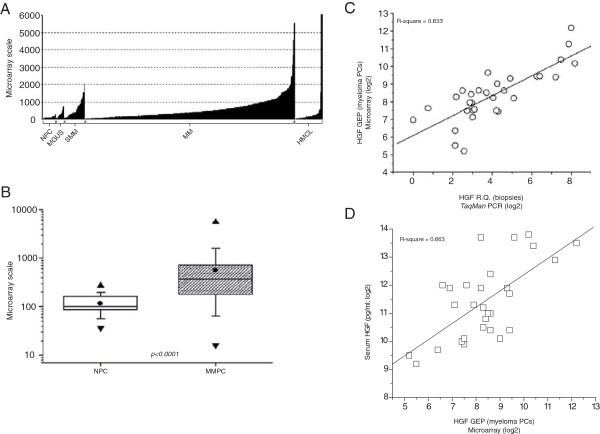
***HGF *gene expression profiles in PCs of healthy individuals and patients suffering from myeloma. (A)** Quantification of HGF mRNA in CD138^+^ cells isolated from bone marrow aspirates. HGF mRNA levels were profiled by the Affymetrix microarray 209961_s_at HGF probe set. Shown are signal strengths obtained by hybridization of mRNA to the microarray. Samples were from healthy individuals (NPC; n = 22); patients with monoclonal gammopathy of undetermined significance (MGUS; n = 14); smouldering multiple myeloma (SMM; n = 34); multiple myeloma (MM; n = 344), and human myeloma cell lines (HMCL; n = 45). **(B)** Statistical analysis of the microarray data from healthy individuals compared to patients with MM. *HGF* gene expression profiles in CD138^+^ cells isolated from bone marrow aspirates of healthy individuals (NPC; n = 22) and MM patients (MMPC; n = 344) were determined by microarray. The result of a two-tailed Mann–Whitney test is shown (p < 0.0001). (●) = mean, (▲) = maximum value, (▼) = minimum value, box = the distribution of the data set, and bars = standard deviation. **(C)** Linear regression analysis of HGF mRNA values measured in bone marrow core biopsies and in CD138^+^ cells isolated from myeloma patients. HGF mRNA in biopsies (n = 46) were quantified by real-time PCR and illustrated as relative quantities (R.Q.). From corresponding patients, CD138^+^ cells (n = 46) were isolated and HGF values were measured by microarray. Shown is the hybridization strength. Values were converted to log2 ratios prior to the linear regression fitting. **(D)** Linear regression analysis of HGF mRNA values in CD138^+^ cells aligned to HGF serum values of corresponding samples. HGF mRNA levels in CD138^+^ cells (n = 29) were determined by microarray. Strength of the hybridization signals are shown. In corresponding samples HGF serum concentrations were measured by ELISA. Quantified serum HGF is shown in pg/mL. Values were converted to log2 ratios prior to the linear regression fitting.

The lack of correlation between HGF mRNA in bone marrow core biopsies and the percentage of MM cells in corresponding samples suggests that HGF is either produced by non-myeloma cells or, if by malignant PCs, that malignant PCs show huge variation between patients in their capacity to produce HGF. To investigate the latter possibility, HGF mRNA expression levels were measured by whole genome cDNA microarray in CD138^+^ cells isolated from bone marrow aspirates of healthy individuals (NPC; n = 22) and patients diagnosed with monoclonal gammopathy of undetermined significance (MGUS; n = 14), smouldering MM (SMM; n = 34), and MM (MM; n = 344). Human myeloma cell lines were also included (HMCL; n = 45) (Figure 2A). From Figure 2A it is obvious that HGF mRNA levels in PCs isolated from bone marrow aspirates vary remarkably within each group. Similar variation in HGF levels has also been described earlier for HGF serum and plasma concentrations [6,7,9]. Detailed analysis of the measured HGF mRNA values in PCs from healthy individuals (NPC; n = 19) and MM patients (MMPC; n = 344) showed statistically significant higher HGF mRNA levels in PCs isolated from MM patients compared to the levels found in CD138^+^ cells of healthy individuals (Figure 2B). Together these data indicate that there is substantial variation in the levels of HGF mRNA produced by malignant plasma cells, and show that CD138^+^ cells are capable of producing high levels of HGF mRNA.

### CD138^+^ cells as the primary source of HGF

As CD138^+^ cells are able of producing large amounts of HGF mRNA, we investigated if these cells are the source of excess HGF. Alignment of the HGF mRNA levels present in the bone marrow core biopsies (n = 46) to the HGF mRNA levels measured in CD138^+^ cells (n = 46) isolated from bone marrow aspirates taken at the same site showed significant correlation (R^2^ = 0.633) as shown in Figure 2C. This indicates that at least in these samples, the PCs are responsible for excess HGF mRNA production. To corroborate this finding, we aligned the *HGF* gene expression profiles (GEP) of CD138^+^ cells (n = 29) to HGF protein concentrations in peripheral blood serum (n = 29) measured in corresponding samples (Figure 2D). Linear regression analysis showed a significant correlation (R^2^ = 0.663) indicating association between HGF mRNA produced by CD138^+^ cells and HGF serum concentrations. In summary these data indicate that the myeloma cells are the primary source of HGF in the bone marrow of myeloma patients with elevated levels of HGF.

### Lack of correlation of HGF mRNA in malignant plasma cells and amplification of *HGF* gene in the same cells

HGF serum values are frequently (approx. 50%) elevated in myeloma patients and a subgroup of myeloma patients, *i.e.* approximately 30%, shows highly elevated HGF serum concentrations. The latter group has a particularly poor prognosis [6], which points to *HGF*-expressing myeloma cells to define this subgroup and raises the question of what the underlying mechanism is which leads to this phenotype.

To see if *HGF* amplifications or translocations could explain the variation in HGF mRNA in malignant plasma cells, we analyzed the number of *HGF* gene copies by FISH (n = 24) and quantified HGF mRNA levels in the same samples by real-time PCR (n = 24). We found that the plasma cells from these patients contained one, two or three copies of *HGF*. As summarized in Table 1, there was no correlation between *HGF* copy number and HGF mRNA levels in these cells. Moreover, we found no evidence of translocations involving *HGF*. Thus, the high HGF mRNA expression in these malignant PC clones is not due to amplifications or translocations of *HGF*. Details of gene copy numbers can be found in Additional file 1: Table S2.

**Table 1:** **Alignment of mRNA levels with the number of *HGF *** **gene copies in corresponding samples**

**Patient no.**	**mRNA levels (R.Q.)**	**No. of *HGF *** **gene copies**
**MM1**	19	3
**MM2**	12	2
**MM3**	36	2
**MM4**	167	1
**MM5**	723	2
**MM6**	546	2
**MM7**	1764	1
**MM8**	617	3
**MM9**	602	2
**MM10**	28	2
**MM11**	N.A.	3
**MM12**	60	N.A.
**MM13**	N.A.	3
**MM14**	903	2
**MM15**	N.A.	2
**MM16**	184	1
**MM17**	155	3
**MM18**	N.A.	2
**MM19**	N.A.	2
**MM20**	N.A.	N.A.
**MM21**	20	N.A.
**MM22**	24	N.A.
**MM23**	1	2
**MM24**	558	N.A.
**MM25**	70	3
**MM26**	200	2
**MM27**	25	2
**MM28**	21	3
**MM29**	8	N.A.
**MM30**	15	3

HGF mRNA levels were quantified by real-time PCR. *HGF* copy numbers were determined by fluorescence *in situ* hybridization.

N.A. – Not available.

R.Q. – Relative quantity.

### Sequencing of the *HGF* promoter region of 12 selected patients

To identify if more subtle changes in *HGF* could explain the differences in HGF mRNA expression, 12 patient samples which were analysed by FISH were further investigated by sequencing of the proximal *HGF* promoter. From the 24 samples analysed by FISH were the 5 samples that showed the lowest HGF serum concentrations (MM 3, 12, 16, 17, 23) and the 7 samples that showed the highest HGF serum concentrations (MM 4, 5, 7, 14, 22, 24, 29) chosen. The *HGF* 5’-UTR of the twelve samples were analyzed by at least three independent overlapping sequencing reactions, considering only high quality sequence traces. The region from approximately −3000 bp to +120 bp relative to the transcription start site (Table 2) was investigated. Despite the large number of SNPs recorded on NCBI dbSNP for this region, only three SNPs were detected in the 12 patients investigated by sequencing [35]. These were Rs3735520, Rs11763015 and Rs149178895 (NCBI dbSNP). Additionally, a homozygotic dC/dT transition at position −1652 (Rs3735520) could be detected in patients MM 7 and MM 24. Apart from that no divergences from the reference sequence were found. In conclusion, there were no obvious mutations in the *HGF* promoter of the myeloma cells investigated that could explain the variation in HGF mRNA expression.

**Table 2:** **Summary of the *HGF *** **promoter sequencing**

**Patient no.**	**SNP (NCBI dbSNP)**	**INDEL mutation**	**NCBI dbSNP**	**DATE length**	**Sequence region**	**HGF Serum levels**
**MM 4**	−1652C/T	–	Rs3735520	27	−2920 – +120	High
**MM 5**	−2142C/A	–	Rs11763015	26	−2870 – +120	High
	−1652C/T		Rs3735520			
**MM 7**	−2142C/A	–	Rs11763015	22	−2910 – -20	High
		−1652C/T	Rs3735520			
**MM 14**	2309 T/A*	–	Rs149178895*	29	−2920 – +120	High
	−1652C/T		Rs3735520			
**MM 22**	–	–		15	−2280 – +120	High
**MM 24**	–	–	Rs3735520	28	−2870 – +120	High
		−1652C/T				
**MM 29**	−1652C/T	–	Rs3735520	27	−2870 – +120	High
**MM 3**	−1652C/T	–	Rs3735520	22	−2870 – +120	Low
**MM 12**	−2142C/A	–	Rs11763015	22	−2840 – -1710	Low
**MM 16**	−1652C/T	–	Rs3735520	23	−2810 – +120	Low
**MM 17**	−1652C/T	–	Rs3735520	26	−2730 – +120	Low
**MM 23**	−2142C/A	–	Rs11763015	25	−2870 – +120	Low
	−1652C/T		Rs3735520			

Genomic DNA isolates from CD138^+^ cells were used to amplify segments of the *HGF* promoter. PCR products were pre-treated with exonuclease I and shrimp alkaline phosphatase and directly used for Sanger DNA sequencing. RefSeq gene NG_016274 was used as reference sequence. SNP and sequence region are relative to transcription start site.

SNP positions Rs3735520 and Rs11763015 are depicted relative to transcription start site (NM_000601.4:c).

*SNP position RS149178895 is depicted relative to gene region NG_016274.1.

### Characterization of deoxyadenosine tract element (DATE) in multiple myeloma patients

**Figure 3:**
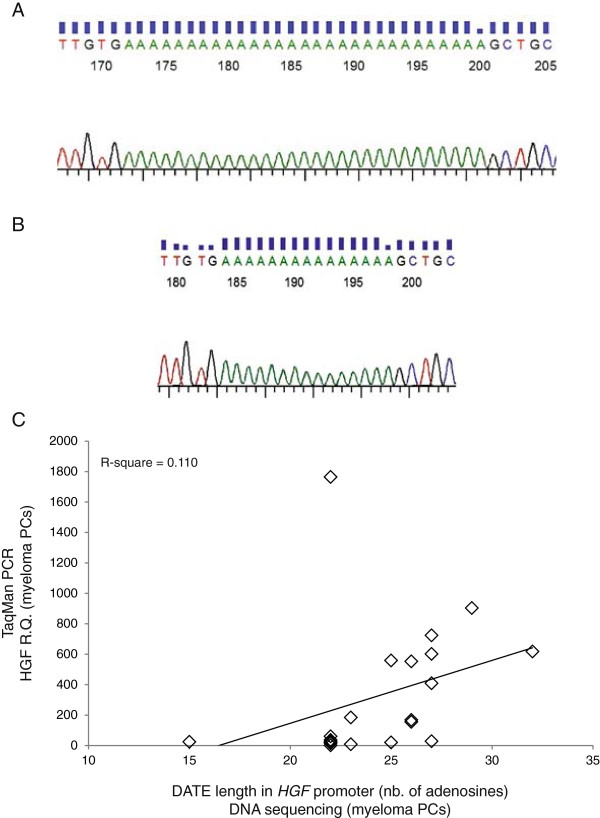
**Characterization of DATE in the *HGF *gene promoter region. (A)** and **(B)** Representative DNA sequencing traces of patients MM 14 and MM 22 with DATEs of 29 and 15 nucleotides, respectively. DATEs of individual patients were amplified by nested PCR, cloned into TA cloning vector pCR2.1 and sequenced using M13 standard primers. **(C)** HGF mRNA levels in CD138^+^ cells are plotted against the number of nucleotides present in DATE of the corresponding samples. CD138^+^ cells purified from bone marrow of myeloma patients were used to quantify HGF mRNA levels by real-time PCR. Corresponding samples were used to isolate genomic DNA for sequencing of DATE in the *HGF* promoter region. Data shown are HGF mRNA mean fold change ± standard deviation and the number of nucleotides quantified by sequencing.

Ma *et al.*[23] described a regulatory deoxyadenosine tract element (DATE) composed of 30 adenosines located about 700 bases upstream of the *HGF* transcription start site (Figure 3A). This element was described to be prone to deletion mutation (Figure 3B). Shortage to less than 25 adenosines was necessary to obtain aberrant *HGF* expression in breast cancer cells and breast tissue, which normally does not express HGF. In contrast, when analyzing the length of DATE in 24 CD138^+^ cell samples isolated from myeloma patient (see Table 2 and Figure 3C), we found no correlation (R^2^ = 0.110) between the length of DATE and HGF mRNA levels in corresponding samples (Figure 3C). The number of adenosines present in DATE varied from 15 to 32 nucleotides, corroborating earlier findings describing DATE to be highly polymorphic [23]. Taken together, the results indicated that there is no correlation between shortening of this poly-adenosine tract in the *HGF* promoter of myeloma cells and the HGF production by the same cells.

### Co-cultivation of bone marrow stromal cells (BMSC) with myeloma cells

On the basis of the above findings we hypothesized that the bone marrow microenvironment might induce elevated HGF production in myeloma cells. To address this we co-cultivated bone marrow stromal cells (BMSC) with various myeloma cell lines for 48 hours, before measuring the produced HGF present in the co-culture supernatant by ELISA.

**Figure 4:**
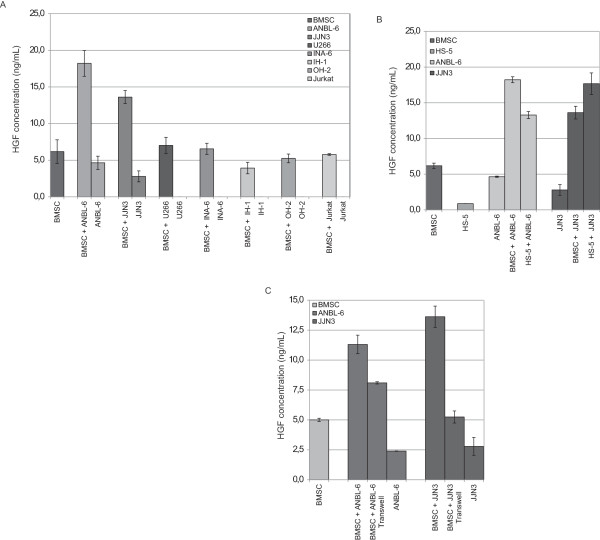
**Production of HGF in co-cultures of BMSC and MM cells.** BMSC or HS-5 cells were seeded at a concentration of 3 × 10^4^ cells per well (0.5 mL) into 24 well plastic plates and 2 – 6 × 10^4^ myeloma cells or the non-myeloma cell line Jurkat were added. Supernatants were harvested after 72 h of incubation. The levels of HGF were measured by enzyme-linked immunosorbent assay. **(A)** BMSC co-cultured with 6 × 10^4^ (ANBL-6, U266, INA-6, IH-1, OH-2 or the non-myeloma cell line Jurkat) or 2 × 10^4^ (JJN3) myeloma cells. **(B)** HS-5 cells co-cultured with 6 × 10^4^ ANBL-6 cells or 2 × 10^4^ JJN3 cells. **(C)** BMSC were seeded into inlet of 24 transwell plastic plates and 6 × 10^4^ ANBL-6 cells or 2 × 10^4^ JJN3 cells per well (0.1 mL) were added to the lower compartment of the transwell. Cultures were maintained as described above, supernatants were harvested after 72 h, and the levels of HGF were measured by enzyme-linked immunosorbent assay.

Co-cultivation of ANBL-6 or JJN3 cells with BMSC led to a significant increase in HGF production (Figure 4A) in the mixed cultures compared to cultures of either cell type alone. U266 cells co-cultured with BMSC also led to a slight, although not significant, increase in HGF production (Figure 4A). Furthermore, the observed effect was not due to changes in cell viability or increased cell proliferation as these factors remained unchanged (data not shown). Co-cultivation of the cell lines IH-1, INA-6 and OH-2 as well as the human T-cell leukemia cell line Jurkat with stromal cells had little or no effect on HGF production.

We also co-cultured ANBL-6 or JJN3 cells with the HS-5 cell line (Figure 4B). Also in this case, co-cultivation led to an increase in secreted HGF comparable to co-culture experiments with BMSC. The HS-5 cell line is an immortalized human bone marrow stromal cell line that produces a number of cytokines such as granulocyte-colony stimulating factor (G-CSF), granulocyte-macrophage-CSF (GM-CSF), interleukin-1α (IL-1α), IL-1β, IL-1RA, IL-6, IL-8, IL-11, but it does not produce significant amounts of HGF (Figure 4B).

To see if cell-cell contact is necessary to obtain this effect, we performed the same experiments, but separated the stromal cells from the myeloma cell lines by transwells. As shown in Figure 4C, co-cultivation of ANBL-6 cells or JJN3 cells with BMSC in transwells also led to an increase in secreted HGF. This effect was however less pronounced as compared to the effect found in co-cultures, suggesting that both soluble factors and cell-cell contacts may lead to increased secretion of HGF.

## Discussion

In myeloma the importance of HGF – c-MET signaling is still unclear although Derksen *et al.* showed that HGF induces proliferation and cell survival in the majority of HMCLs investigated and in about 50% of malignant PCs isolated from myeloma patients [4,5]. In a different study investigating the efficacy of a c-MET inhibitor it was shown that in a HGF dependent cell line as well as in primary CD138^+^ cells inhibition of the HGF – c-MET signaling pathway induces cell death and counter acts the proliferative potential induced by HGF [36]. These findings provide strong evidence that HGF – c-MET signaling might be of importance for myeloma cell survival at least in the subpopulation of myeloma patients which have high levels of HGF in the blood serum. The source of elevated HGF levels in these patients is still unclear. We therefore investigated its origin and found that HGF mRNA levels were significantly elevated in bone marrow core biopsies of myeloma patients if compared to mRNA values in biopsies of healthy individuals. This, together with the fact that there was no association between the measured HGF mRNA levels and the proportion of malignant PCs present in the specimens investigated, suggests that *HGF* is overexpressed in the bone marrow of myeloma patients. This is in agreement with the findings made by Mahtouk *et al.* who showed by performing a microarray study that *HGF* is expressed in both malignant PCs and in cells of the BM microenvironment, but not in healthy PCs [12]. HGF values in plasma and peripheral blood serum are frequently elevated, *i.e.* in approximately 50% of patients [6,7]. Also the measured HGF protein and HGF mRNA levels in malignant PCs show large variations, as we and others have shown in previous reports [12-14]. Our *HGF* gene expression profiling data reflect these findings as the observed HGF mRNA levels measured in CD138^+^ cells isolated from bone marrow aspirates of healthy individuals and myeloma patients at different disease stages varied widely within each sample group. Interestingly, despite the large sample range, HGF mRNA levels in malignant PCs were significantly elevated if compared to HGF mRNA in CD138^*+*^ cells isolated from healthy individuals. This is corroborated by a microarray study performed by Zhan *et al.*, were they found *HGF* to be the only cytokine that was upregulated in myeloma cells as compared to PCs of healthy individuals [12,20]. Investigating the role of myeloma cells in the production of HGF, we found a clear correlation not only between HGF mRNA levels in malignant PCs and bone marrow core biopsies of myeloma patients, but also between HGF mRNA levels and HGF serum concentrations of corresponding samples. Collectively, these findings strongly suggest CD138^+^ cells as the main source of excess HGF in myeloma patients. An investigation of whether HGF protein levels in patient sera and HGF protein levels in corresponding CD138^+^ cell samples correlate or not would further strengthen these findings, however such experiments could not be performed as CD138^+^ cells are available only in limited numbers and become apoptotic when cultured for an extended time period.

Investigating the molecular mechanisms behind the HGF production in myeloma cells, we looked for genetic aberrations possibly responsible for the excess HGF production. However, we found no apparent mutations or amplifications of the HGF gene that could explain elevated HGF production. To clarify if a mutation could be responsible for excess HGF, we sequenced the *HGF* promoter region of CD138^+^ cells isolated from twelve patients. No point mutations could be detected. Despite the large number of SNPs described for the sequenced region, only three SNPs were present, *i.e.* Rs3735520, Rs11763015 and Rs149178895. SNP Rs149178895 is located at position 2309 based on the *HGF* reference sequence (NG_016274.1), was found only once in patient MM 14, and two patients were homozygous for a dC to dT transitional mutation at position −1652 (Rs3735520) relative to the transcription start site. SNP Rs3735520 was found to associate with end-stage lung disease in Japanese systemic sclerosis patients, and carriers of the *HGF* promoter with the *HGF −*1652 TT allele had a relative inability to increase circulating HGF levels. By functional studies, the *HGF* promoter carrying the *HGF −*1652 TT allele was reported to have lower transcriptional activity than the promoter carrying the CT or CC allele, possibly due to the binding of a negative transcriptional regulator [37]. In myeloma, the relevance of these SNPs, in particular Rs3735520, needs further clarification. However, it cannot be ruled out that the limited number of SNPs detected is due to the small sample size analyzed and the regional and ethnical restriction of the sample collection.

In breast cancer, a negative regulatory deoxyadenosine tract element (DATE) composed of about 30 adenosines was described to be present in the HGF promoter. This element was described to be important for gene silencing in breast tissue. If DATE becomes shortened by at least five adenosines, the element loses its repressive effect resulting in HGF expression in breast tissue and breast cancer cells [23]. We also found this element to be highly polymorphic and prone to deletion mutation [23,38]. Alignment of DATE length to HGF serum concentrations or HGF mRNA levels did not show any correlation. This is in contrast to earlier findings where DATE was found to lose its negative regulatory effect upon deletion of at least 5 adenosines [23,38]. However, the power of this analysis needs to be questioned due to the limited number of samples investigated. More importantly, on the basis of these findings it is unlikely that genetic aberrations in the HGF gene are responsible for the elevated HGF serum levels.

The absence of mutations that could explain the high *HGF* expressing phenotype of certain myeloma cell clones points to other mechanisms that induce excessive HGF production. We therefore investigated if the bone marrow microenvironment could induce excessive HGF production in certain myeloma cells. By performing co-cultivation experiments of BMSC with various myeloma cell lines we found that co-cultures of stromal cells with ANBL-6 cells or JJN3 cells led to a marked increase in HGF production. The co-cultivation of BMSC with U266 cells led as well to an increased HGF production, although this effect was not significant.

The increase in HGF production in co-cultures of myeloma cells with stromal cells could also be seen with different types of stromal cells. The BMSC applied here are mainly fibroblast-like cells obtained from the CD138 negative fraction of bone marrow mononuclear cells isolated from myeloma patients. The HS-5 cells are a fibroblastic cell line immortalized with papilloma virus genes E6 and E7. These cells were found to support proliferation of hematopoietic progenitor cells without the need for exogenous factors [27]. Cultivation of myeloma cell lines separated from BMSC by a permeable membrane in transwell experiments showed that soluble factors alone are sufficient for increased HGF production. However the combination of soluble factors and cell-cell contacts led to the most pronounced increase of secreted HGF. This possibly reflects the dependence of myeloma cells on their microenvironment. These findings suggest exogenous factors present in the bone marrow microenvironment to be of importance for *HGF* overexpression in malignant PCs.

Interestingly, we observed increased HGF production in co-cultures of BMSC with the cell lines ANBL-6 and JJN3, but not with other myeloma cell lines. Both ANBL-6 and JJN3 cells produce substantial amounts of HGF also when cultured alone. This indicates that stromal cells are only able to induce elevated HGF production in myeloma cells which already are capable of making HGF.

## Conclusions

In this study, we investigated the origin of elevated HGF levels found in myeloma patients. The main result presented here is that in multiple myeloma malignant plasma cells are the prime source for the high HGF levels in the bone marrow and peripheral blood serum. As shown in previous studies, HGF protein levels in plasma and serum from myeloma patients show large variations and we here confirm this on the mRNA level measured in CD138^+^ cells. Furthermore, we demonstrated that neither cytogenetic aberrations of the *HGF* gene, nor mutations in the *HGF* promoter, but rather exogenous factors present in the bone marrow microenvironment lead to increased HGF production by malignant PCs. The data showing that BMSCs increase HGF production in myeloma cells is consistent with the view that myeloma cells largely rely on and respond to the bone marrow niche. These findings might help to identify a subpopulation of myeloma patients which suffer from a myeloma cell clone that produces large amounts of HGF. This could be of value in clinical applications, and in particular in relation to the bone disease of multiple myeloma, where elevated HGF levels associate with severity of disease.

## Abbreviations

BMSC: Bone marrow stromal cells; DATE: Deoxyadenosine tract element; ELISA: Enzyme-linked immunosorbent assay; FISH: Fluorescence *in situ* hybridization; HGF: Hepatocyte growth factor; HMCL: Human myeloma cell line; MGUS: Monoclonal gammopathy of undetermined significance; MM: Multiple myeloma; NPC: Non-myeloma plasma cell; PC: Plasma cell; R.Q.: Relative quantity; SMM: Smouldering multiple myeloma.

## Competing interests

The authors declare that they have no competing interests.

## Authors' contributions

CR, ET, TKV and GB performed experiments. CR and ET designed the research and analyzed the data. AW provided samples and reviewed the work. TSS gave suggestions, proofread and reviewed the manuscript. MB and AS supervised the research and provided funding. All authors have read and approved the final manuscript.

## Supplementary Material

Additional file 1: Table S1 Primers for the amplification of the HGF promoter, DATE nested PCR primers and sequencing primers. **Table S2.** Cytogenetic status of the patient samples investigated by fluorescence *in situ* hybridization. Click here for file
