# Supplementary material for: Identification of the source of elevated hepatocyte growth factor levels in multiple myeloma patients
Source: Biomark Res. 2014 Apr 9;2:8. doi: 10.1186/2050-7771-2-8 (PMC4022385; doi:10.1186/2050-7771-2-8)
Supplement: Additional file 1: Table S1 — Primers for the amplification of the HGF promoter, DATE nested PCR primers and sequencing primers. Table S2. Cytogenetic status of the patient samples investigated by fluorescence in situ hybridization. [file 2050-7771-2-8-S1.pdf]

Supplementary material

Supplementary Table 1: Primers for the amplification of the *HGF* promoter, DATE nested PCR primers and sequencing primers

**Amplification Primers**			
**2.0kpHGF For**	5′-	GGAACATGTGATTCTTCTCCTCGTGGGGT	-3′
**3.4kpHGF Rev**	5′-	TGTCAGCCTCTGTCTGGTGTCCCC	-3′
**3.3kpHGF For**	5′-	TCAGGGTCACGGTCAGGCTATGC	-3′
**4.7kpHGF Rev**	5′-	GGCACAGGCAGGGGACCAATTCGC	-3′
**3.2kpHGF For**	5′-	CAGAGAGAGCTGATTCTGAGGTCTTC	-3′
**4.3kpHGF Rev**	5′-	CTGCCAAACTCACGAAATACCACAC	-3′
**HGF_DATEoutFor**	5′-	GTCCAAAGCAGCCAAGTTCAGCCAG	-3′
**5.5kpHGF Rev**	5′-	GGGACAGGCTATGGACAATGACTGTTTCTTGG	-3′

**DATE nested Primers**		As described in (19)	
**HGF_DATEoutFor**	5′-	GGGACAGGCTATGGACAATGACTGTTTCTTGG	-3′
**HGF_DATEoutRev**	5′-	GGGTGTGGTATTGTGGGGCCAAAATAAG	-3′
**HGF_DATEinFor**	5′-	CGTGAGTTTGGCAGTTTGTG	-3′
**HGF_DATEinRev**	5′-	ATGGGCTCAGAGCAGGCAGC	-3′

**Sequencing Primers**			
**2.7kpHGFSeq1**	5'-	ACCCGAGAGCCTTTAGAAC	-3'
**2.7kpHGFSeq2**	5'-	TCATTTTTCTGAGCCCCTAC	-3'
**2.7kpHGFSeq3**	5'-	AGTTACCAGAGCATCCACC	-3'
**pHGF-5-Seq**	5'-	GGTGAAAGTCAGTCCTAACCAG	-3'
**pHGF-Seq3**	5'-	GTGCCTTGATTTAGCCATTG	-3'
**pHGFSeqRev2**	5'-	TGGCTAAATCAAGGCACAG	-3'
**pHGFSeqRev3**	5'-	AACAGTCATTGTCCATAGCC	-3'
**pHGFSeqRev4**	5'-	CCAAAGTGTAAGTGGAAAGATGAGG	-3'
**pHGFSeqRev5**	5'-	TTGTCAGCCTCTGTCTGGTGTCC	-3'
**pHGFSeqRev6**	5'-	CCATTCCAAAGAAGGTCC	-3'

Supplementary Table 2: Cytogenetic status of the patient samples

Patient	Age at sampling	Gender	Hyperdiploidy		Other Cytogenetics	Treatment status	% PC in BM
MM 1	55	M	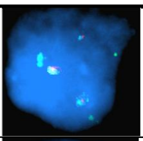	Three copies *HGF*<br>Three copies *CEP7*	N.A.	Newly Diagnosed / Untreated	17
MM 2	74	M	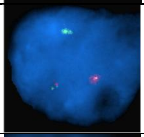	Two copies *HGF*	Normal *IGH*, del17<br>Positive del13	Newly Diagnosed / Untreated	66
MM 3	65	M	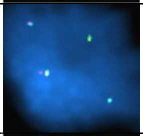	Two copies *HGF*	Normal *IGH*, del13 and del17	Newly Diagnosed / Untreated	60
MM 4	81	M	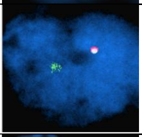	One copy *HGF*	t(14;16)<br>Normal del13 and del17	Newly Diagnosed / Untreated	95
MM 5	72	M	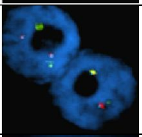	Two copies *HGF*	Normal *IGH*, del13, del17, *IGK*, *IGL*	Newly Diagnosed / Untreated	60
MM 6	77	M	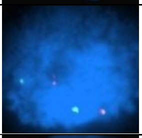	Two copies *HGF*	t(11;14)<br>Normal del13<br>Normal del17, 1p & gain 1q	Newly Diagnosed / Untreated	20
MM 7	65	F	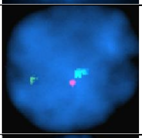	One copy *HGF*	t(11,14)<br>Positive del13<br>Normal del17	Newly Diagnosed / Untreated<br>MGUS diagnosed in 2004	45
MM 8	72	M	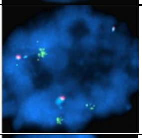	Three copies *HGF*	Normal *IGH* & del17<br>Positive del13<br>Normal 1p & gain 1q	Diagnosed in August 2006 / Treated	>95
MM 9	81	M	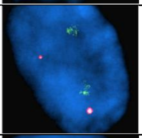	Two copies *HGF*	Normal *IGH*, del13, del17, 1p & 1q	N.A.	N.A.
MM 10	62	M	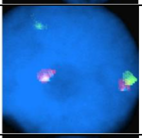	Two copies *HGF*	t(11;14)<br>Positive del13<br>Normal del17, 1p & 1q	Newly Diagnosed / Untreated	40
MM 11	73	M	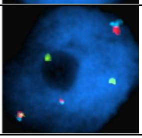	Three copies *HGF*	Normal *IGH*, del17, 1p & 1q<br>Positive del13<br>*IGK* split	Newly Diagnosed / Untreated	>30
MM 12	67	M	N.A.	N.A.	t(4;14), t(2;8)<br>Positive del13<br>Normal del17	Newly Diagnosed / Untreated	5-10
MM 13	71	M	N.A.	Three copies *HGF*	Normal *IGH*, del17, 1p & 1q<br>Positive del13	Newly Diagnosed / Untreated	28

Supplementary Table 2: Continued

Patient	Age at sampling	Gender	Hyperdiploidy		Other Cytogenetics	Treatment status	% PC in BM
MM 14	81	F	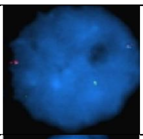	Two copies *HGF*	Unknown *IGH* split<br>Positive del13<br>Normal del17, 1p & 1q	Newly Diagnosed / Untreated	60
MM 15	56	M	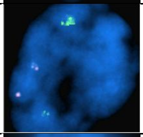	Two copies *HGF*	Normal *IGH*, del13, del17, 1p & 1q	Newly Diagnosed / Untreated	31
MM 16	63	M	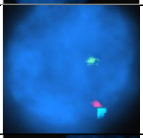	One copy *HGF*	Normal *IGH*, del13 and del17	Newly Diagnosed / Untreated	8
MM 17	57	M	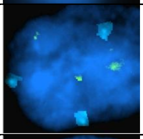	Three copies *HGF*	Normal *IGH*, del13, *MYC*, 1p & gain 1q	Newly Diagnosed / Untreated	5
MM 18	80	F	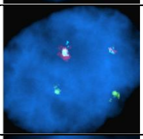	Two copies *HGF*	Normal *IGH*, del 17, 1p, 1q, *CEP15*<br>Positive del13<br>Triploid 9	Diagnosed in 2005 / Untreated	13
MM 19	67	F	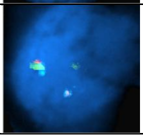	Two copies *HGF*	t(11;14)<br>Positiv del13<br>Normal del17, 1p & gain 1q	Newly Diagnosed / Untreated	10
MM 20	80	F	N.A.	N.A.	Normal *IGH*<br>del17, *MYC*, 1p, 1q, *CEP9*, *CEP19*, positiv del13	Newly Diagnosed / Untreated	44
MM 21	56	M	N.A.	N.A.	t(4;14)<br>Positiv del13 and del17, normal 1p gain 1q	Newly Diagnosed / Untreated	7
MM 22	53	M	N.A.	N.A.	Unknown *IGH* split<br>Normal del13 and del17	N.A.	N.A.
MM 23	60	M	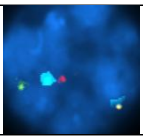	Two copies *HGF*	Unknown *IGH* split<br>Positive del13 and del17<br>Normal 1q & 1q	N.A.	N.A.
MM 24	68	M	N.A.	N.A.	t(11;14)<br>Normal del13 and del17, deletion 1p & gain 1q	N.A.	N.A.
MM 25	72	M	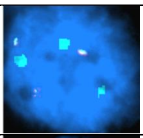	Three copies *HGF*<br>Three copies *CEP7*	t(11;14)<br>Normal del13<br>del17, 1p & 1q	N.A.	N.A.
MM 26	36	M	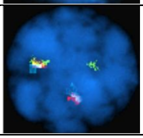	Two copies *HGF*	t(4;14)<br>Positive del13<br>Normal del17, 1p & 1q	Newly Diagnosed / Untreated	N.A.

Supplementary Table 2: Continued

Patient	Age at sampling	Gender	Hyperdiploidy		Other Cytogenetics	Treatment status	% PC in BM
MM 27	53	F	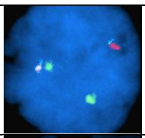	Two copies *HGF*	t(11;14)<br>Positive del13<br>del17, del 1p, Normal 1q	Cells isolated after first CyDex treatment / Serum withdrawn after ASCT	N.A.
MM 28	59	M	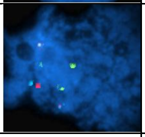	Three copies *HGF*	Unknown *IGH* split<br>Normal del13<br>Positive del17	N.A.	N.A.
MM 29	66	F	N.A.	N.A.	t(11;14)<br>Normal del13<br>del17,1p & 1q	N.A.	N.A.
MM 30	58	M	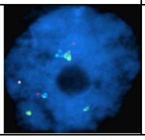	Three copies *HGF*	t(11;14)<br>Normal del13<br>del17,1p & 1q	N.A.	N.A.

M	Male
F	Female
BM	Bone Marrow
N.A.	Not Analyzed
PC	Plasma cells
CyDex	Cyclophosphamide and Dexamethasone treatment (proceeds ASCT)
ASCT	Autologous Stem Cell Therapy
